# Different MicroRNA Families Involved in Regulating High Temperature Stress Response during Cotton (*Gossypium hirsutum* L.) Anther Development

**DOI:** 10.3390/ijms21041280

**Published:** 2020-02-14

**Authors:** Jin Chen, Ao Pan, Shujun He, Pin Su, Xiaoling Yuan, Shengwei Zhu, Zhi Liu

**Affiliations:** 1 College of Bioscience and Biotechnology, Hunan Agricultural University, Changsha 410128, China; jinchen_0704@163.com (J.C.); panao1216@163.com (A.P.); yuanxiaolingcs@163.com (X.Y.); 2 Cotton Sciences Research Institute of Hunan Province, Changde 415101, China; heshujun1975@126.com; 3 Hunan Plant Protection Institute, Hunan Academy of Agricultural Sciences, Changsha 410125, China; supin102@163.com; 4 Key laboratory of Plant Molecular Physiology, Institute of Botany, Chinese Academy of Sciences, Beijing 100093, China

**Keywords:** cotton, high temperature stress, miRNA family, anther development, miRNA sequencing

## Abstract

MicroRNAs (miRNAs) are small molecule RNAs widely involved in responses to plant abiotic stresses. We performed small RNA sequencing of cotton anthers at four developmental stages under normal and high temperature (NT and HT, respectively) conditions to investigate the stress response characteristics of miRNA to HT. A total of 77 miRNAs, including 33 known miRNAs and 44 novel miRNAs, were identified, and 41 and 28 miRNAs were differentially expressed under NT and HT stress conditions, respectively. The sporogenous cell proliferation (SCP), meiotic phase (MP), microspore release period (MRP), and pollen maturity (PM) stages had 10 (including 12 miRNAs), four (including six miRNAs), four (including five miRNAs), and seven (including 11 miRNAs) HT stress-responsive miRNA families, respectively, which were identified after removing the changes in genotype-specific miRNAs under NT condition. Seven miRNA families (miR2949, miR167, and miR160 at the SCP stage; miR156 and miR172 at the MP stage; miR156 at the MRP stage; and miR393 and miR3476 at the PM stage), which had expression abundance of more than 10% of the total expression abundance, served as the main regulators responding to HT stress with positive or negative regulation patterns. These miRNAs orchestrated the expression of the corresponding target genes and led to different responses in the HT-tolerant and the HT-sensitive lines. The results revealed that the HT stress response of miRNAs in cotton anthers were stage-specific and differed with the development of anthers. Our study may enhance the understanding of the response of miRNAs to HT stress in cotton anthers and may clarify the mechanism of plant tolerance to HT stress.

## 1. Introduction

Cotton (*Gossypium hirsutum* L.) is one of the most economically important crops because its fiber is a raw material used by the textile industry worldwide. During the whole process of growth and development, the cotton plant is sensitive to temperature, and the ideal temperature range is 20–30 °C. At present, the human population growth and global industrialization are the driving forces of the accelerating global warming. The direct manifestation of climate change is the rise in global temperatures, especially the frequent occurrence of extreme high temperature (HT) during summer, which occurs at the same time as the flowering period of cotton. Long-term HT stress may result in anther indehiscence, microspore abortion, and premature degeneration of the tapetum [1], which eventually leads to boll shedding, threatening the global cotton production. HT stress decreases pollen fertility and crop yield, but the molecular mechanism of the effect of HT stress on plant pollen fertility is still unknown.

MicroRNAs (miRNAs) are a class of small endogenous RNA molecules derived from longer single-stranded precursors (pre-miRNA) [2,3]. The length of mature miRNAs range between 20 and 24 nucleotides (nt). The miRNAs ubiquitously exist in the plant kingdom, and are highly conserved in all kinds of organisms like mosses, flowering monocots, and dicots [4]. miRNAs negatively regulate the expression of target genes at the post-transcriptional regulation by degrading target mRNAs or repressing gene translation [5].

For almost a decade, some miRNAs have been categorized into different groups (miRNA families) on the basis of mature miRNA, seed sequence (the positions 2–8 nt from the miRNA 5ʹ-end) [6], and/or structure of pre-miRNAs [7,8]. A miRNA family is an important unit in the classification of miRNA functions because the sequence and function are conserved among the members of miRNAs within the same family [2,9]. Many plant miRNAs are species-specific, however, those functionally important to plant development and environment adaption are conserved across different species [10]. The species-specific miRNAs are not usually in an organ- or tissue-specific pattern, and tend to evolve neutrally [10,11]. Consequently, these species-specific miRNAs obtained stable and specialized functions [12,13].

miRNAs are involved in the plant’s response to abiotic stresses [4], such as drought, salinity, low temperature (LT), and HT [14,15]. Currently, many miRNAs have been detected in plants, including rice, wheat, barley, and *Arabidopsis,* as a response to heat stress [16,17,18,19]. miR156 was the first miRNA identified in plants and plays a critical role in plant phase change and heat stress memory [20,21]. Under heat stress conditions, miR156 is highly induced [20,22], and plants overexpressing miR156 exhibit enhanced tolerance to heat stress [20,21]. HT stress inhibits the expression of miR156 in cotton [15], the miR157, which belongs to the same miRNA family as that of miR156, shows the same expression trend. Male infertility is observed in miR157-overexpressing plants, suggesting that the target genes of the same family of miRNAs are functionally identical or similar [6,23], and the induced expression of miR157 partially compensates for the loss of function due to the inhibition of miR156 expression. miR172 is involved in the development of anthers in many species, such as *Arabidopsis*, cotton, and alfalfa [15,24,25]. miR172 regulates floral organ identity genes, such as *AP2* and *AP2*-like genes (*TOE, TOE2,* and *SMZ*) [24], and its expression is considerably inhibited under HT stress, whereas its target gene, *TOE*, is upregulated [16].

At present, only a few miRNAs have been reported to be involved in cotton anther, and the complete picture of miRNAs or miRNA families and their roles during cotton anther development under HT stress need further investigation. In this study, the miRNA profiles of HT-sensitive and HT-tolerant cotton lines were compared, and the miRNAs and the miRNA family at the whole-genome level were analyzed. Moreover, the HT-responsive miRNA families at different developmental stages of cotton anthers were identified. Results showed that the miRNA family responding to HT stress was significantly different at different developmental stages. The miR160, miR167, and miR2949 families are the main HT stress-responsive families at the sporogenous cell proliferation (SCP) stage. The miR156 and miR172 families showed high abundance ratio at the meiotic phase (MP). Only the miR156 family was dominantly expressed at the microspore release period (MRP). Lastly, miR3476 and miR393 were the major families that responded to HT stress at the pollen maturity (PM) stage. The putative target genes of these major HT stress-responsive miRNA families were predicted from the transcriptome sequencing data, and a model of the miRNA-mediated HT stress response during cotton anther development was further established by combining the mRNA-seq and the small RNA (sRNA)-seq information. The identified HT stress-responsive miRNAs and their targets can help illuminate the mechanisms of HT stress tolerance in cotton.

## 2. Results

### 2.1. Global Analysis of sRNA Sequencing

Based on the statistical analysis of sequencing results, the 16.8 M clean reads can be measured for each sample on average, and the average ratio of the mapping rate was above 96% (Table S1). The sequences from the anthers constituted four groups according to the four developmental stages (Figure S1), the early stages (SCP and MP) and the late stages (MRP and PM) were clustered together, respectively (Figure S2). The clean and unique reads in the early stages of anther development were 148.2 and 51.56 M, whereas those in the late stages of anther development were 120.4 and 30.81 M, respectively (Figure S3). The read length statistics revealed that all samples showed the highest abundance at 24 nt, followed by 21 nt (Figure S4a). Usually, a 24 nt sRNA is considered a small interfering RNA (siRNA) involved in the RNA-directed DNA methylation [26,27]. The change in the ratio of 24 to 21 nt (24 nt/21 nt) may reflect the degree of DNA methylation. Results showed that the 24 nt/21 nt in the late stages was remarkably lower than in the early stages. T4 had the lowest ratio (2.6), and H1 had the highest ratio (2.9 times that of T4) (Figure S4b).

Clean reads were aligned against the miRBase. Novel miRNAs were predicted for sequences that lacked homology to known miRNAs and the “-novel” was added in the standard name to distinguish from known miRNAs. A total of 77 miRNAs, including 33 known miRNAs and 44 novel miRNAs (seven novel miRNAs cannot be uniformly named), were identified in all samples from the four anther developmental stages (miRNAs with TPM (transcripts per million) < 10 in all samples were removed) (Table S2). The number of miRNAs detected in the early stages of anther development was more than that in the late stages (Figure S5).

All 70 miRNAs (33 known and 37 novel miRNAs) were classified into 41 miRNA families. Among these, 13 families contained two or more members, and the rest were single-member families. The miR482 family contained seven members, followed by the miR3476 and miR156 families, each including five members (Table S2). The seven unnamed miRNAs belonged to seven upland cotton-specific miRNA families, indicating that species-specific miRNAs also exist under the premise of high homology of miRNAs.

### 2.2. Identification and Expression Abundance Analysis of Differentially Expressed miRNAs

**Figure 1:**
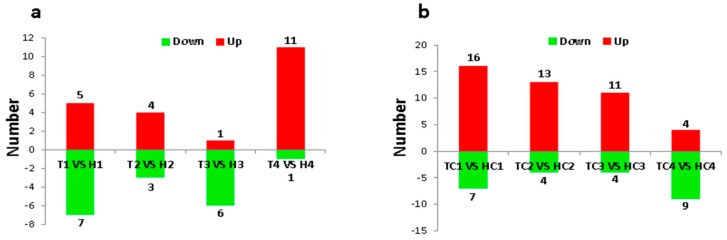
Histograms of differentially expressed microRNAs (miRNAs) under normal temperature (NT) and high temperature (HT) conditions. (**a**) The number of differentially expressed miRNAs in the anthers between HT-tolerant line and the HT-sensitive line under HT stress at sporogenous cell proliferation (SCP) (H1 vs. T1), meiotic phase MP (H2 vs. T2), microspore release period MRP (H3 vs. T3), and pollen maturity (PM) (H4 vs. T4) stages. (**b**) The number of differentially expressed miRNAs in the anthers between HT-tolerant line and the HT-sensitive line under NT condition at SCP (HC1 vs. TC1), MP (HC2 vs. TC2), MRP (HC3 vs. TC3), and PM (HC4 vs. TC4) stages.

A total of 68 miRNAs (44 upregulated and 24 downregulated) were differentially expressed under normal temperature (NT) condition (Figure 1b). While comparing the HT-tolerant line and the HT-sensitive line at each anther developmental stage, only 38 differentially expressed miRNAs (21 upregulated and 17 downregulated) were found under HT stress (Figure 1a). Redundant miRNAs that appeared at different stages were removed, and 41 and 28 unique differentially expressed miRNAs were identified under NT and HT conditions, respectively.

**Figure 2:**
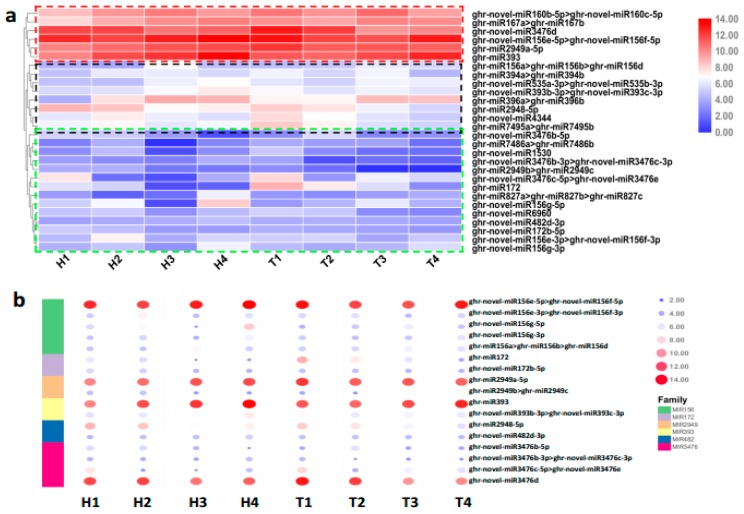
Differential expression of miRNAs at the four stages of anther development under HT stress. (**a**) Heatmap of the 28 differentially expressed miRNAs responding to HT stress. miRNAs in red-, black-, and green-dotted boxes have high, medium, and low expression levels, respectively. (**b**) Heatmap of six major miRNA families responding to HT stress.

According to the expression level, 6, 8, and 14 differentially expressed miRNAs had high, moderate, and low abundance, respectively, under HT stress considering all the developmental stages (Figure 2a). All 28 differentially expressed miRNAs under HT stress belonged to 17 miRNA families, which contained six multimember miRNA families (miR156, miR172, miR2949, miR393, miR482, and miR3476). The interesting point is that each multimember family had only one member with high abundance (TPM ≥ 500), and the others had low abundance (TPM < 500) (Figure 2b). Two types of miRNAs generally showed opposite expression trends in the anthers of the HT-sensitive and the HT-tolerant lines under HT stress (Figure 2b, Table S3). Additionally, the single-member families miR160 and miR167 had high expression abundance, and the remaining miRNAs (miR535, miR394, miR396, miR827, miR1530, miR4344, miR6960, miR7486, and miR7495) had low expression abundance.

### 2.3. Various miRNA Families Responding to HT Stress at Different Developmental Stages

**Figure 3:**
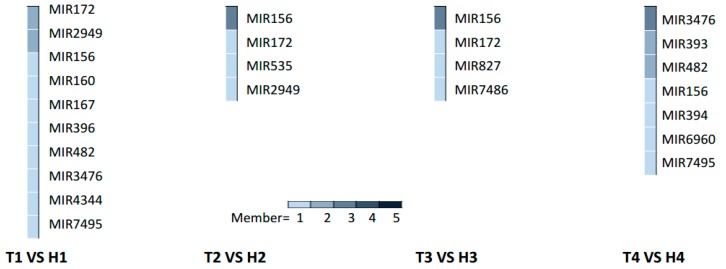
Anther stage-specific miRNA families responding to HT stress in the HT-tolerant and the HT-sensitive lines.

The HT stress-responsive miRNA families at different developmental stages were obtained from the comparative analysis of differentially expressed miRNAs in the HT-tolerant and the HT-sensitive lines under HT treatment after removing the changes in genotype-specific miRNAs under NT condition (Figure 3 and Figure S6). The SCP, MP, MRP, and PM stages had 10 (including 12 miRNAs), four (including six miRNAs), four (including five miRNAs), and seven (including 11 miRNAs) HT stress-responsive miRNA families, respectively (Figure 3). This result illustrated that the SCP stage had more miRNAs or miRNA families that responded to HT stress than the other three stages.

At the SCP stage, the multimember miR172 and miR2949 families showed significantly differential expression patterns in the HT-tolerant and the HT-sensitive lines under HT stress. The high abundance of family members in the two families (ghr-miR172 and ghr-miR2949a-5p) were downregulated in the anther of the HT-tolerant line, whereas the other family members (ghr-novel-miR172b-5p and ghr-miR2949b > ghr-miR2949c) showed opposite expression trends (Table S3). The single-member families, namely, miR160, miR396, miR7495, miR4344, and miR167, were downregulated in the HT-tolerant line after suffering from HT stress, whereas the other single-member families miR156, miR482, and miR3476 were upregulated.

The miR156 family responded to HT stress at the MP and the MRP stages. The expression trends of ghr-novel-miR156e-3p > ghr-novel-miR156f-3p and ghr-miR156a > ghr-miR156b > ghr-miR156d increased and decreased, respectively, at the MP stage. The ghr-novel-miR156e-5p > ghr-novel-miR156f-5p was remarkably induced by HT stress at the MRP stage. The expression trend of ghr-novel-miR156g-3p was suppressed in the HT-tolerant line. The ghr-novel-miR156g-5p, a differentially expressed miRNA shared by the MP and the MRP stages, showed opposite expression trends in the two periods. At the MP stage, ghr-novel-miR156g-5p was substantially induced in the HT-tolerant line, but its expression was inhibited by HT stress at the MRP stage (Table S3). Other families (miR172, miR535, miR827, and miR7486) were suppressed in the HT-tolerant line except for miR2949, which was upregulated at the MP stage.

At the PM stage, the members of the miR3476, miR482, and miR393 families also showed opposite expression patterns in the HT-sensitive and the HT-tolerant lines after HT treatment. ghr-miR393, ghr-miR2948-5p, and ghr-novel-miR3476d were upregulated in the HT-tolerant line, whereas ghr-novel-miR393b-3p > ghr-novel-miR393c-3p, ghr-novel-miR482d-3p, ghr-novel-miR3476b-5p, ghr-novel-miR3476b-3p > ghr-novel-miR3476c-3p, and ghr-novel-miR3476c-5p > ghr-novel-miR3476e were downregulated. Four families (miR394, miR156, miR7495, and miR6960) in the anthers of the HT-tolerant line were induced by HT stress (Table S3).

**Figure 4:**
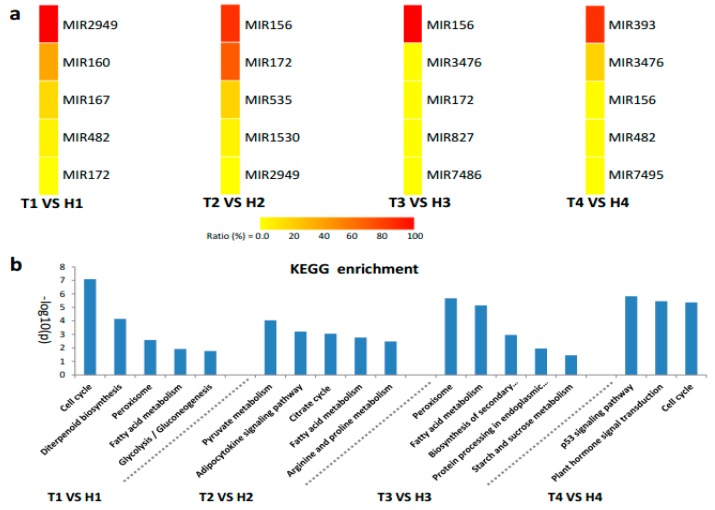
Heatmap of the expression abundance of differentially expressed miRNA families and Kyoto Encyclopedia of Genes and Genomes (KEGG) enrichment analysis of the predicted target genes. (**a**) The top five expression abundance rates of miRNA families responding to HT stress in the HT-tolerant and the HT-sensitive lines. The expression abundance rate is determined as the expression abundance (in percentage) of a miRNA family over the total expression abundance of miRNA families at a certain stage (sum of family member’s expression abundance/the sum of expression abundance of all differentially expressed miRNAs at a certain stage). (**b**) KEGG enrichment of target genes of miRNA families with high expression abundance.

**Figure 5:**
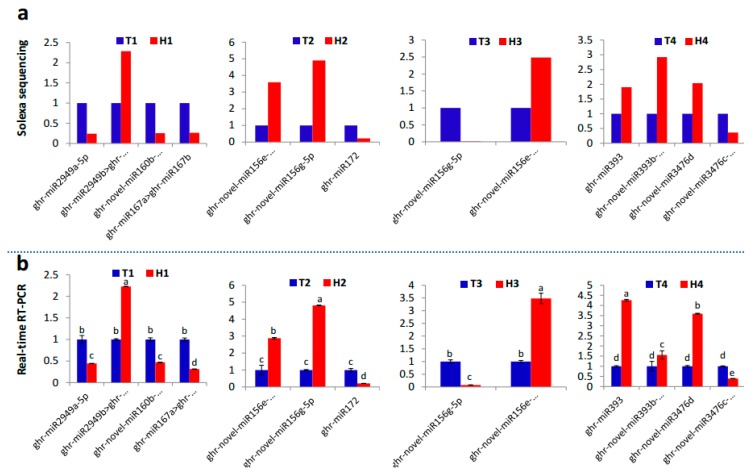
qRT-PCR validation of the main HT responsive miRNAs identified by small RNA sequencing. (**a**) Fold-change of miRNAs at the four developmental stages under HT stress based on small RNA sequencing data. (**b**) The relative expression of the same miRNA members under HT stress detected by qRT-PCR and represented as means of three replicates (*n* = 3) ± standard error. Significant differences indicated with letters above each bar (*p* ≤ 0.05).

The identified miRNA families and their diverse expression patterns at different stages implicated the complexity of miRNAs involved in the regulation of the response of anthers to heat stress. Usually, the miRNAs with high abundance are considered the dominant and functional products that respond to stresses, such as HT [28,29]. In our study, seven miRNA families (miR2949, miR167, and miR160 at the SCP stage; miR156 and miR172 at the MP stage; miR156 at the MRP stage; and miR393 and miR3476 at the PM stage) with expression abundance of more than 10% of the total expression abundance served as the main regulators that respond to HT stress with positive or negative regulation patterns (Figure 4a). The expression of the members from these miRNA families at different developmental stages under HT stress were confirmed by qRT-PCR, and the expression trend was highly similar with the results of small RNA sequencing (Figure 5).

### 2.4. Target Gene Prediction and Functional Enrichment

The main HT stress-responsive miRNA families at different developmental stages were used to predict the target genes to understand the possible biological functions or mechanistic pathways of miRNAs (Table S4). The functions and annotations were analyzed using the Kyoto Encyclopedia of Genes and Genomes (KEGG) enrichment (Figure 4b). At the SCP stage, 656 target genes were mainly annotated to cell cycle, fatty acid metabolism, and peroxisomes. The KEGG enrichment results revealed that 562 and 221 predicted target genes at the MP and the MRP stages, respectively, were concentrated on energy metabolism. This finding suggested that energy supply was very important during the early development of anther in response to HT stress. In addition, the genes involved in the metabolism of peroxisomes, arginine, and proline and protein processing at the two critical stages can scavenge the reactive oxygen species (ROS) and denatured proteins generated by HT stress to maintain normal anther development. At the PM stage, the functions of 50 target genes were enriched on cell cycle, plant hormone signal transduction, and p53 signaling pathway, all of which are necessary for next-step pollination and responding to stress stimuli.

**Figure 6:**
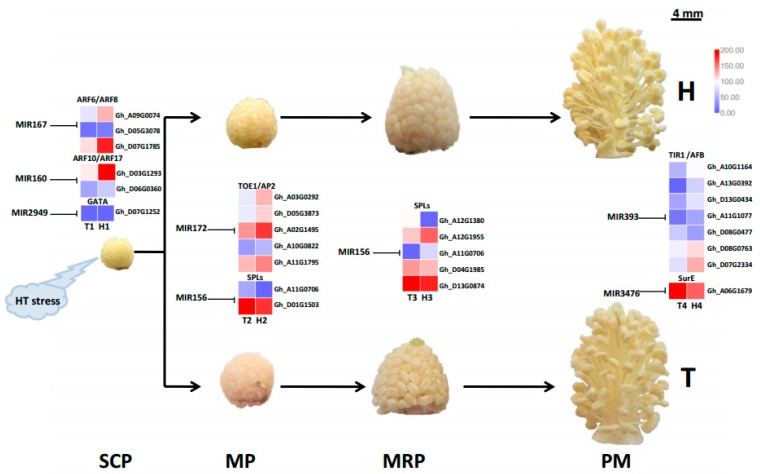
A schematic diagram showing various miRNA families and their target genes involved in cotton male sterility caused by HT stress at different stages of anther development.

**Figure 7:**
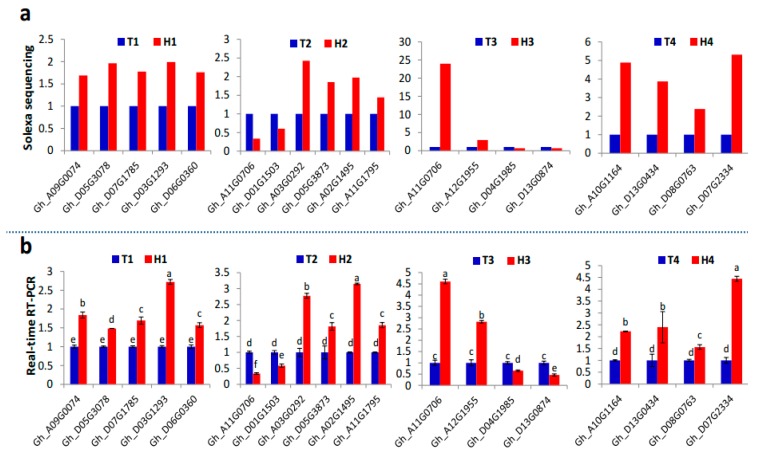
qRT-PCR validation of 19 predicted target genes identified by transcriptome sequencing. (**a**) Fold-change of the predicted genes at the four developmental stages under HT stress based on transcriptome sequencing data. (**b**) The relative expression of the same genes under HT stress detected by qRT-PCR and represented as means of three replicates (*n* = 3) ± standard error. Significant differences indicated with letters above each bar (*p* ≤ 0.05).

All predicted target genes were further searched and aligned against the differentially expressed genes identified from the transcriptome sequencing data at four anther developmental stages in the HT-tolerant and the HT-sensitive lines under HT stress (Table S5). Results showed that *ARF6* (Gh_A09G0074 and Gh_D05G3078), *ARF8* (Gh_D07G1785), *ARF10* (Gh_D03G1293), *ARF17* (Gh_D06G0360), and *GATA* (Gh_D07G1252) were the target genes of miR167, miR160, and miR2949 families at the SCP stage. miR156-target *SPLs* participated in HT stress response at the MP and MRP stages. However, the different *SPL* genes at the MP (Gh_A11G0706 and Gh_D01G1503) and the MRP (Gh_A12G1380, Gh_A12G1955, Gh_A11G0706, Gh_D04G1985, and Gh_D13G0874) stages demonstrated diverse expression patterns in the HT-tolerant and the HT-sensitive lines because significantly different expression profiles existed among the three members of the miR156 family. In addition, miR172 was inhibited by HT stress at the MP stage. Its target genes *TOE1* (Gh_A03G0292 and Gh_D05G3873) and *AP2* (Gh_A02G1495, Gh_A10G0822, and Gh_A11G1795) were dramatically induced in the anthers of the HT-tolerant line. At the PM stage, the target genes *TIR1* (Gh_A10G1164, Gh_A13G0392, Gh_D13G0434, Gh_A11G1077, Gh_D08G0477, and Gh_D08G0763) and *AFB* (Gh_D07G2334) were identified in the miR393 family, and *SurE* (Gh_A06G1679) was identified in the miR3476 family. The main HT stress-responsive miRNA families and their target genes at different anther developmental stages are summarized in the schematic diagram shown in Figure 6. The expression of 19 predicted genes targeted by the identified HT responsive miRNAs (Figure 5) under HT stress were further confirmed by qRT-PCR (Figure 7), and the expression trends (upregulation or downregulation) of these target genes were highly consistent between the data of transcriptome sequencing and qRT-PCR (Figure 7). The expression and regulation of these miRNA families and target genes fluctuated differently between the HT-tolerant and the HT-sensitive lines during anther development. This fluctuation ultimately led to indehiscent, shrunken, and empty anthers in the HT-sensitive line under HT stress.

## 3. Discussion

### 3.1. More miRNAs Participate in the Early Period of Anther Development

The regulation of genomic DNA methylation involves 24 nt siRNAs [26,27]. The changes in the 24 nt/21 nt may reflect changes in the variation of DNA methylation during anther development. The increased frequency of genomic DNA methylation inhibits the expression of miRNAs and mRNAs [27,30]. However, in our study, a high level of 24 nt/21 nt was in parallel with a high number of reads (clean and uniq reads) in the early stages of anthers (Figures S3 and S4b). The same results were detected in miRNAs regardless whether they are HT-tolerant or HT-sensitive lines or in NT or HT conditions (Figure S5). More miRNAs during the early stage of anthers may indicate that a strong post-transcriptional regulation occurred, which may involve nucleic acid and protein syntheses and meiosis [5] These results showed that the early stage of anther development may be an important period for miRNAs to participate in the regulation of anther development.

### 3.2. The Expression of High- and Low-Abundance miRNAs Changes Diversely in Response to HT Stress

The number of differentially expressed miRNAs under NT condition was remarkably higher than that under HT stress, but no difference was found in the categories of miRNAs detected at both temperature conditions. It is speculated that miRNAs have achieved HT stress response by regulating the expression abundance (upregulation or downregulation) of existing miRNAs in cells rather than inducing the expression of new types of miRNA [31].

Each multimember family, which was derived from a family classification of differentially expressed miRNAs under HT stress, contained one member with high abundance and one or more members with low abundance (Figure 2b). The expression trends of these two types of miRNAs were opposite in the HT-sensitive and the HT-tolerant lines (Figure 2b, Table S3). The miRNAs within the same family have a similar function attributable to their conserved sequence and structural configuration [6,23]. High- and low-abundance miRNAs may play major and minor regulatory roles, respectively [28,29]. The coordinated regulation of these two types of abundance of miRNAs ultimately leads to the regulation of heat stress response [28].

### 3.3. Different MiRNA Families Temporally and Elaborately Regulate Anther Development under HT Stress

The differentially expressed miRNAs in the HT-sensitive and the HT-tolerant cultivars showed a period of specificity in family classification under HT stress (Figure 3). This discovery implied that the different stages of anther development were regulated by different miRNA families.

The miR160, miR167, and miR2949 families showed high abundance ratio expressions at the SCP stage under HT stress (Figure 4a). The miR160 and miR167 families are involved in HT stress response by regulating their target gene *ARF* [16]. Overexpressing miR160 increased cotton sensitivity to HT stress, as shown by anther indehiscence, which was associated with the suppression of *ARF10* and *ARF17* expressions [15]. The KEGG enrichment of target genes showed that the three high-abundance miRNA families were mainly involved in the energy metabolic pathways, such as glycolysis, gluconeogenesis, and fatty acid synthesis (Figure 4b). In addition, some target genes were enriched in diterpenoid biosynthesis and in peroxisome and cell cycle signaling pathways (Figure 4b). At the SCP stage, sporulated cells proliferate in the anthers while the anthers are preserving large amounts of energy [5]. In this strategy, high-abundance miRNAs in the HT-tolerant line, including the members of the miR160, miR167, and miR2949 families (ghr-novel-miR160b-5p > ghr-novel-miR160c-5p, ghr-miR167a > ghr-miR167b, and ghr-miR2949a-5p) were downregulated (Figure 5), contributed to the accumulation of energy substances in cells, and ensured the proliferation of pollen mother cells. However, certain concentrations of secondary metabolites and fatty acids can increase the tolerance of cells to HT [32,33]. The SCP anthers suffered from HT stress, which can disturb cell energy metabolism and redox balance in the HT-sensitive line and eventually lead to male sterility.

The miR156 family had high abundance ratios during the MP and the MRP periods. Furthermore, the miR172 family exhibited high abundance expression during the MP period (Figure 4a). miR156 is involved in cell division and differentiation regulation in the early stage of anther development [34]. miRNA156 can be significantly induced by HT stress, leading to male sterility [21,22,35]. The overexpression of miR156 in *Arabidopsis* causes ambient temperature-sensitive flowering [22]. In this study, ghr-novel-miR156g-3p and ghr-novel-miR156g-5p were significantly induced in the MRP stage of the HT-sensitive line (Figure 5), which may explain the phenotypic changes in the T-line under HT stress. The miR172 family is involved in multiple species, such as *Arabidopsis*, cotton, and alfalfa, and well known for its response to HT stress [15,24,25]. Under HT stress, the expression of miRNA172 is inhibited [35,36,37]. The conversion of sepals to petals into carpels and reduction of stamen number were achieved by overexpression of miRNA172 [24]. In this study, ghr-miR172 was significantly downregulated in the anther of the HT-tolerant line (Figure 5).

The target gene prediction and the functional enrichment analysis showed that the target genes of high-abundance miRNA families were involved in energy metabolism at the MP and the MRP stages (Figure 4b). Moreover, a part of the target genes was involved in peroxisome synthesis, arginine and proline metabolism, and biosynthesis of secondary metabolites at the MP and the MRP stages (Figure 4b). The MP and the MRP stages are meiosis and microspore release periods, respectively, during which cells need to consume large amounts of energy to ensure normal meiosis and maturation of pollen grains [5]. However, vigorous respiration produces a large amount of ROS, and the synthesis of peroxisomes, arginine, and proline can effectively remove excess ROS in cells and prevent oxidative damage [38]. Meiosis is the period that is most sensitive to heat stress [39,40,41]. The accumulation of secondary metabolites in cells helps increase the heat tolerance of plants under HT stress [33,42]. Heat stress tolerance is a comprehensive process that requires multiple factors to achieve a dynamic balance. Possibly, the abnormal expression of the miR156 and miR172 families disrupted this balance and eventually caused the T-line to exhibit HT sensitivity.

At the last stage of anther development, the miR393 and miR3476 families showed high abundant expression at the same time (Figure 3). The members of the two families (ghr-miR393 and ghr-novel-miR3476d) were upregulated in the HT-tolerant line (Figure 5). miR393 can be induced by a variety of abiotic stresses, such as HT or LT and drought [35,43,44]. Currently, the function of the miR3476 family has not been reported, suggesting that the response mechanism of anthers to HT stress may exceed our current knowledge.

The functional enrichment results showed that the target genes of miR393 and miR3476 families were involved in cell cycle, plant hormone signal transduction, and the p53 signaling pathways (Figure 4b). The cell cycle pathway was presumed to be related to the transformation of mononuclear pollen grains to binuclear pollen grains. HT stress can considerably affect the expression of p53 pathway genes [45], which are involved in a variety of cellular functions, such as apoptosis, growth inhibition, and cellular stress aging [45,46]. Furthermore, endogenous phytohormones, such as auxin, gibberellin, and jasmonate (JA), have been reported to respond to HT stress during anther development [15,47,48].

### 3.4. Various MiRNA Families and Their Target Genes Generate Cotton Male Sterility under HT Stress

The miRNAs participate in regulation of plant reproduction development and abiotic stress responses [49]. miR160 and miR167 are responsive to HT stress through their target genes (*ARF*s), which are involved in the auxin signaling pathway [18,19]. miR172 is a well-studied miRNA [50,51] that targets *AP2*-like genes, such as *TOE1*, *TOE2*, and *SMZ* [50]. These target genes are important transcription factors during the differentiation of floral organs [52]. *TOE1* interacts with a subset of JAZ proteins and promotes the biosynthesis of JA, resulting in male sterility in *Arabidopsis* [53,54]. Multiple *AP2* genes were upregulated in the HT-tolerant line, especially Gh_D05G3873, Gh_A03G0292, Gh_A02G1495, and Gh_A11G1795 (Figure 6 and Figure 7). HT stress may have affected anther development by regulating the expression of miR172.

*SPLs*, as target genes of miR156, are essential for anther development and male fertility in many plants [55,56]. The upregulation of miR156 in HT-sensitive line suppressed the expression of *SPLs*, which increased the sensitivity of anthers to HT stress. In *Arabidopsis*, miR156-mediated *SPL* downregulation has increased the plant’s response to environmental stresses [20]. In this study, two differentially expressed *SPLs* (Gh_A11G0706 and Gh_D01G1503) were downregulated in the HT-tolerant line at the MP stage (Figure 6 and Figure 7). At the MRP stage, five *SPLs* were differentially expressed (two were upregulated, and three were downregulated), and Gh_A11G0706 showed remarkably differential expression in the MP and MRP stages. Interestingly, Gh_A11G0706 was significantly induced in the HT-tolerant line from the MP stage to the MRP stage, whereas the opposite trend was observed in the HT-sensitive line (Figure 6 and Figure 7). However, *SPLs* act as a transcriptional activator of miR172, which prevents the expression of *AP2* [50,51]. HT stress was proposed to inhibit the expression of *SPLs* in the HT-sensitive line, leading to the accumulation of *AP2*, which interacted with the JAZ protein. The excessive accumulation of JA ultimately leads to male sterility [53,54].

*TIR1* and *AFB* are important phytohormone auxin receptors involved in the response to HT stress as target genes of miR393 [14,57,58]. At the PM stage, HT stress inhibited the expression of *TIR1* and *AFB* in the HT-sensitive line (Figure 6 and Figure 7) and promoted the excessive accumulation of auxin, which may ultimately lead to male sterility [5].

## 4. Materials and Methods

### 4.1. Plant Materials

The cotton (*Gossypium hirsutum* L.) lines used in this research included an HT-tolerant line HLY11 (H) and an HT-sensitive line TS18 (T) [59]. The cotton seeds were sown and grown in greenhouses with a normal temperature (NT) condition, which was characterized by a 12 h/12 h photoperiod and a 28–34 °C/20–28 °C day/night thermoperiod. For the high-temperature treatment, the temperature condition was set to a 39 ± 2 °C/29 ± 2 °C day/night thermoperiod that lasted for 10 d when the cotton plants were flowering (approximately 60 d after sowing).

The buds at the four developmental stages, namely, SCP, MP, MRP, and PM stages, were harvested under NT and HT conditions [5]. The anther tissue of each sample was isolated, immediately frozen in liquid nitrogen, and stored at −70 °C until use for transcriptomic and sRNA sequencing. After HT stress treatment, the anthers of TS18 were indehiscent, shrunken, and empty, whereas those of HLY11 were split normally with abundant pollen grains. No difference was observed between the two cotton lines under NT condition (Figure S7).

### 4.2. RNA Extraction and Sequencing

Total RNA was extracted from the materials using the TRIzol reagent kit (Invitrogen, Carlsbad, CA, US) following the manufacturer’s instructions. The RNA yield was determined using the NanoDrop 2100 spectrophotometer (Thermo Scientific, Waltham, MA, USA). Partial RNA for transcriptome sequencing was obtained using the Illumina HiSeqTM2500 (OE biotech company, Shanghai, China). The rest of the RNAs were subjected to gel electrophoresis. The fragments with a certain size were recovered, and 3ʹ- and 5ʹ-end linkers were added. The platform of the Illumina HiSeq XTEN was adopted to obtain the sRNA sequencing (OE biotech company, Shanghai, China). The sequencing data were deposited in the NCBI Sequence Read Archive (http://www.ncbi.nlm.nih.gov/Traces/sra, accessed on 22, August, 2019) with accession number SRP218740.

### 4.3. Data Analysis

The reference genome and transcriptome link of upland cotton were obtained from ftp://public.genomics.org.cn/BGI/cotton/Gossypium_hirsutum/Gossypium_hirsutum_v1.0.gz, accessed on 1, July, 2018. A comparative analysis of the cotton reference genome was made for clean bases [60]. Clean reads were normalized using the following formula: normalized expression = mapped read count/total reads*1,000,000. The uniform screening conditions for the differential expression of mRNA and miRNA were *p* ≤ 0.05 and fold change ≤ 0.5 or fold change ≥ 2. The comparison between the sequence in this study and the body sequence of the mature miRNA in miRbase (used as know miRNA) was carried out with the Bowtie software [61]. The miRDeep2 software was adopted to predict the novel miRNA by combining the homologous miRNA sequence of cotton and the RNA secondary structure prediction software like RNA fold [62]. An online prediction website (http://mongrn.org/psRNATarget/, accessed on 18, October, 2019) was used to perform the target gene prediction. The target genes were deciphered via the Kyoto Encyclopedia of Genes and Genomes (KEGG) analysis [63]. A heatmap was drawn using the TBtools software (South China Agricultural University, Guangzhou, China) [64].

### 4.4. Real-Time Quantitative Polymerase Chain Reaction (PCR)

Three biological replicates were obtained from each sample for real-time PCR analysis to detect miRNA and mRNA abundance under NT and HT conditions. The primer pairs (Table S6) used for RT-PCR were designed via the Roche LCPDS2 software and synthesized via the Generay PCR (Generay Biotech, Shanghai, China).

The RT-PCR of mRNA was described in a previous study [65]. The miRNAs quantification was achieved by a two-step reaction method, which consisted of reverse transcription (RT) and PCR. The reaction agents for each RT reaction was 0.5 μg RNA, 2 μL miScript HiSpec buffer, 1 μL Nucleics Mix, and 0.5 μL miScript reverse transcriptase mix (Qiagen, Dusseldor, Germany). The volume for each reaction was 10 μL, and the reactions were performed using the GeneAmp^®^ PCR System 9700 (Applied Biosystems, Foster City, CA, USA) with the following conditions: 60 min at 37 °C, followed by heat inactivation of RT for 5 min at 95 °C. The 10 μL RT reaction mix was then diluted 10 fold in nuclease-free water and stored at −20 °C. The real-time PCR was performed using the Light Cycler^®^ 480 II RT-PCR instrument (Roche, Basel, Swiss) with 10 μL PCR reaction mixture that included 1 μL cDNA, 5 μL 2× QuantiFast^®^ SYBR^®^ Green PCR Master Mix (Qiagen, Dusseldor, Germany), 0.2 μL universal primer (Qiagen, Dusseldor, Germany), 0.2 μL microRNA-specific primer, and 3.6 μL nuclease-free water. Reactions were incubated in a 384-well optical plate (Roche, Swiss) at 95 °C for 5 min, followed by 40 cycles at 95 °C for 10 s and at 60 °C for 30 s. At the end of the PCR cycles, melting curve analysis was performed to validate the specific generation of the expected PCR product.

The actin (NM 001327051) gene was used as reference gene to normalize the expression level of mRNAs, and the 5S genes were used for miRNAs. The expression amounts were calculated using the comparative 2^−ΔΔCt^ method [66].

## 5. Conclusions

In conclusion, sRNA libraries were established at different developmental stages of cotton anthers under HT stress and NT conditions by using high-throughput sequencing technology. A total of 77 miRNAs, including 33 known miRNAs and 44 novel miRNAs, were identified. A total of 41 and 28 miRNAs were differentially expressed under NT and HT conditions, respectively. After removing the changes in genotype-specific miRNAs under NT condition, SCP, MP, MRP, and PM stages had 10 (including 12 miRNAs), four (including six miRNAs), four (including five miRNAs), and seven (including 11 miRNAs) HT stress-responsive miRNA families, respectively. Seven miRNA families with expression abundance of more than 10% of the total expression abundance at the four stages of cotton anther development were the main and important regulators that respond to HT stress with positive or negative regulation patterns. Overall, our results will enhance the understanding of the response of miRNAs to HT stress in cotton anthers.

## Supplementary Materials

Supplementary materials can be found at https://www.mdpi.com/1422-0067/21/4/1280/s1. Figure S1. Principal component analysis (PCA) of clean reads in anthers at four developmental stages under NT and HT conditions. The 16 samples include the anthers from the HT-tolerant line (H) and the HT-sensitive line (T) under NT (controls) condition at SCP (HC1 and TC1), MP (HC2 and TC2), MRP (HC3 and TC3), and PM (HC4 and TC4) stages and anthers from the HT-tolerant line and the HT-sensitive line under HT stress at SCP (H1 and T1), MP (H2 and T2), MRP (H3 and T3), and PM (H4 and T4) stages. Figure S2. Cluster analysis of clean reads in anthers at four developmental stages under NT and HT conditions. Figure S3. Statistics of clean and unique reads in the early and late stages of anther development. Figure S4. Length distribution and 24 nt/21 nt sRNAs. (a) Length distribution of 18–26 nt sRNAs in 16 samples. (b) The 24 nt/21 nt sRNAs in 16 samples. Figure S5. Total number of miRNAs in the early and late stages of anther development in the HT-tolerant and the HT-sensitive lines under NT (HC and TC) and HT (H and T) conditions, respectively. Figure S6. Family of differentially expressed miRNAs at four anther developmental stages in the HT-tolerant and the HT-sensitive lines under HT (a) and NT (b) conditions. The red marked miRNA families, including miR1530 at the MP stage, miR3476 and one member of miR156 at the MRP stage, and miR827 at the PM stage, presented the same expression patterns and were considered genotype-specific miRNAs. Figure S7. Morphological observation of cotton anther at the dehiscence period under NT and HT conditions. Anthers from the HT-tolerant (HLY11) and the HT-sensitive (TS18) lines under NT (a and b, respectively) and HT (c and d, respectively) conditions. Table S1. Small RNA sequencing data and miRNA statistics in 16 samples, including the anthers from the HT-tolerant line (H) and the HT-sensitive line (T) under NT (controls) condition at SCP (HC1 and TC1), MP (HC2 and TC2), MRP (HC3 and TC3), and PM (HC4 and TC4) stages and anthers from the HT-tolerant line and the HT-sensitive line under HT stress at SCP (H1 and T1), MP (H2 and T2), MRP (H3 and T3), and PM (H4 and T4) stages. Table S2. All miRNAs detected by sRNA sequencing (miRNAs with TPM < 10 in all samples were removed), miRNA sequences, expression levels (TPM) in each sample, standard name of known and novel miRNAs, and family classification. Table S3. Differentially expressed miRNAs under HT stress. Table S4. List of predicted target genes. Table S5. List of differentially expressed genes. Table S6. List of primers for qRT-PCR.
