# Supplementary figures and images for: Different MicroRNA Families Involved in Regulating High Temperature Stress Response during Cotton (Gossypium hirsutum L.) Anther Development
Source: Int J Mol Sci. 2020 Feb 14;21(4):1280. doi: 10.3390/ijms21041280 (PMC7072957; doi:10.3390/ijms21041280)

PCA 3D figure

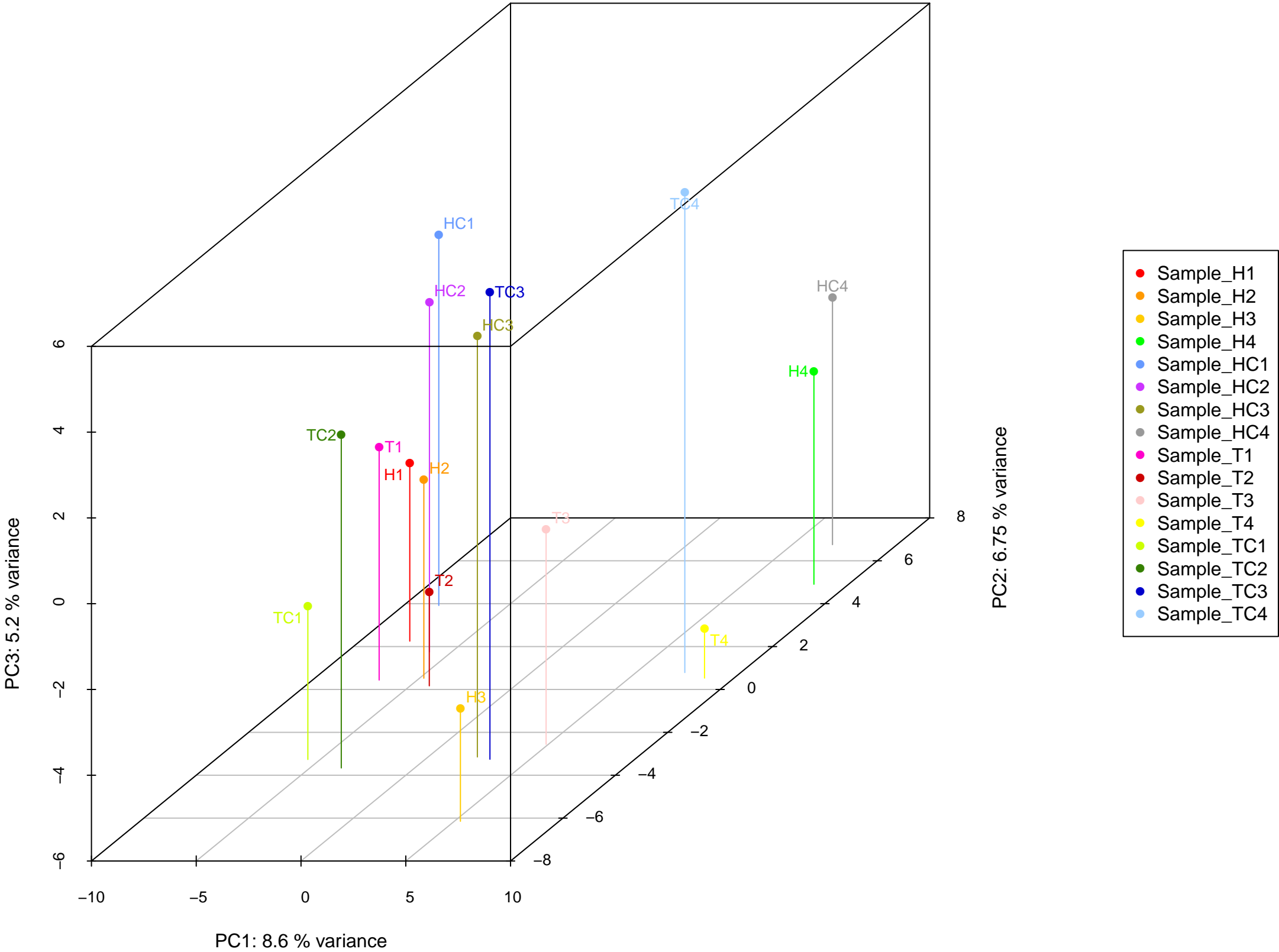

Supplement: Supplementary file 1 [file ijms-21-01280-s001.zip › Supplementary files/Supplementary Figure S1.pdf]

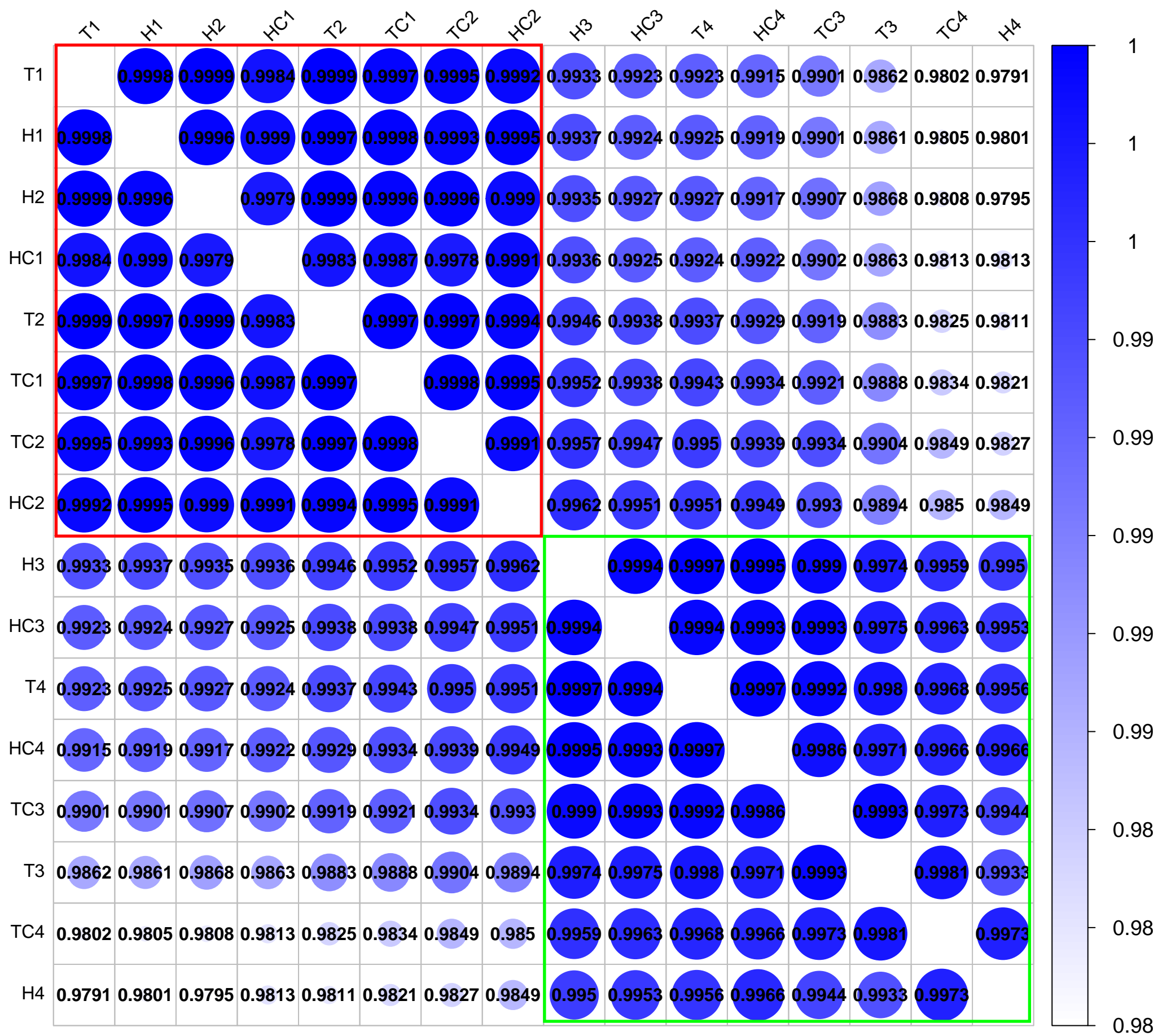

Supplement: Supplementary file 1 [file ijms-21-01280-s001.zip › Supplementary files/Supplementary Figure S2.pdf]

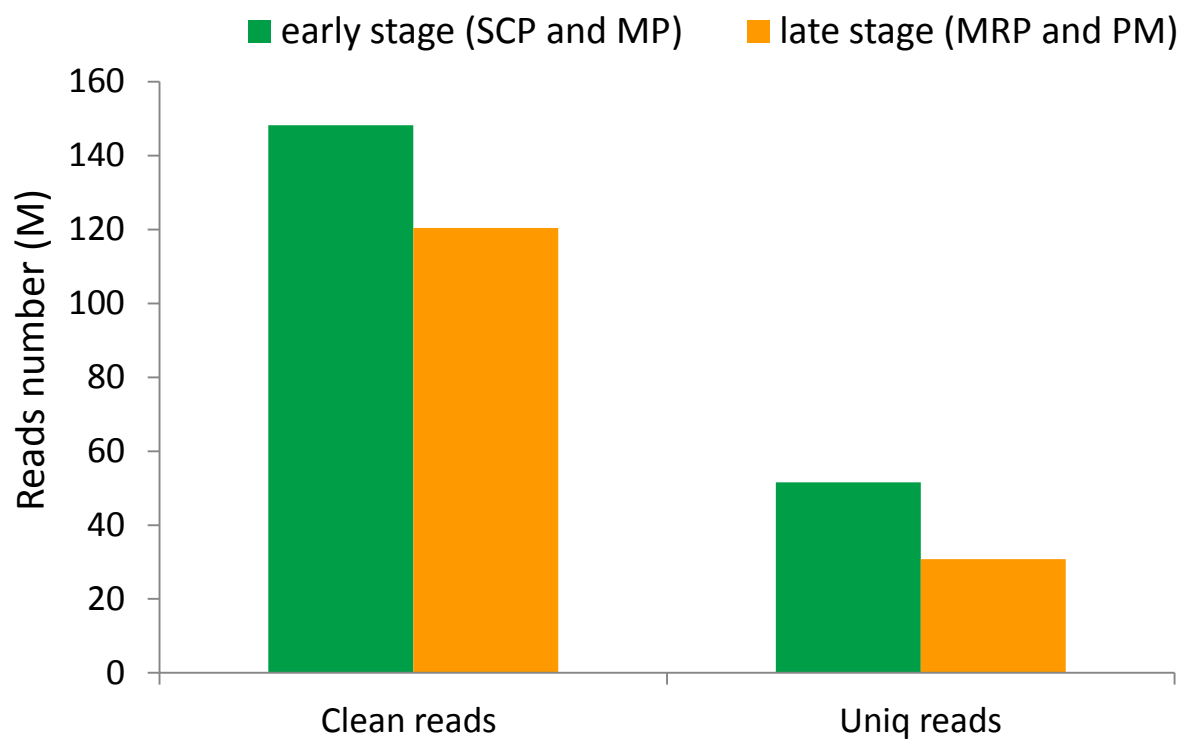

Supplement: Supplementary file 1 [file ijms-21-01280-s001.zip › Supplementary files/Supplementary Figure S3.pdf]

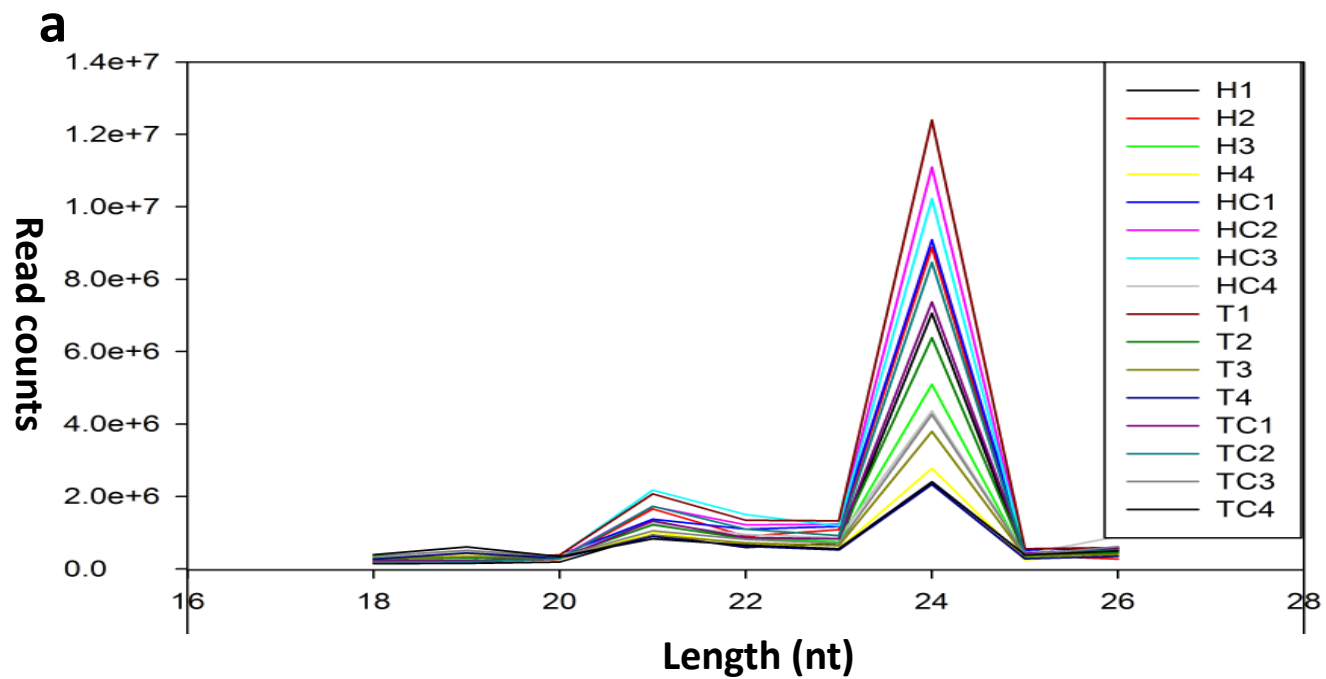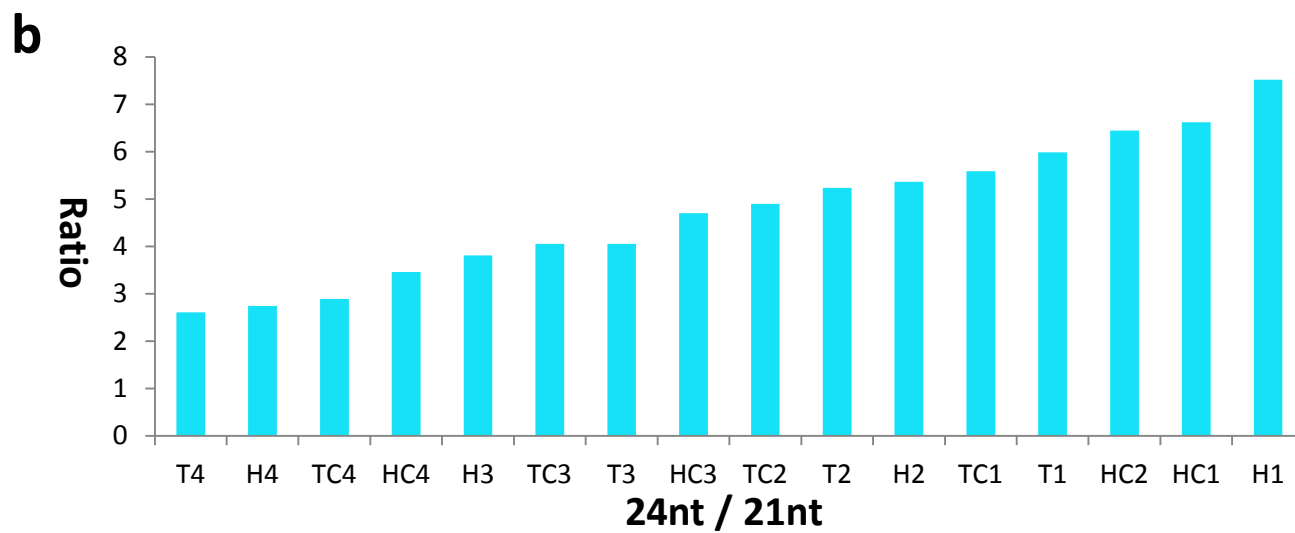

Supplement: Supplementary file 1 [file ijms-21-01280-s001.zip › Supplementary files/Supplementary Figure S4.pdf]

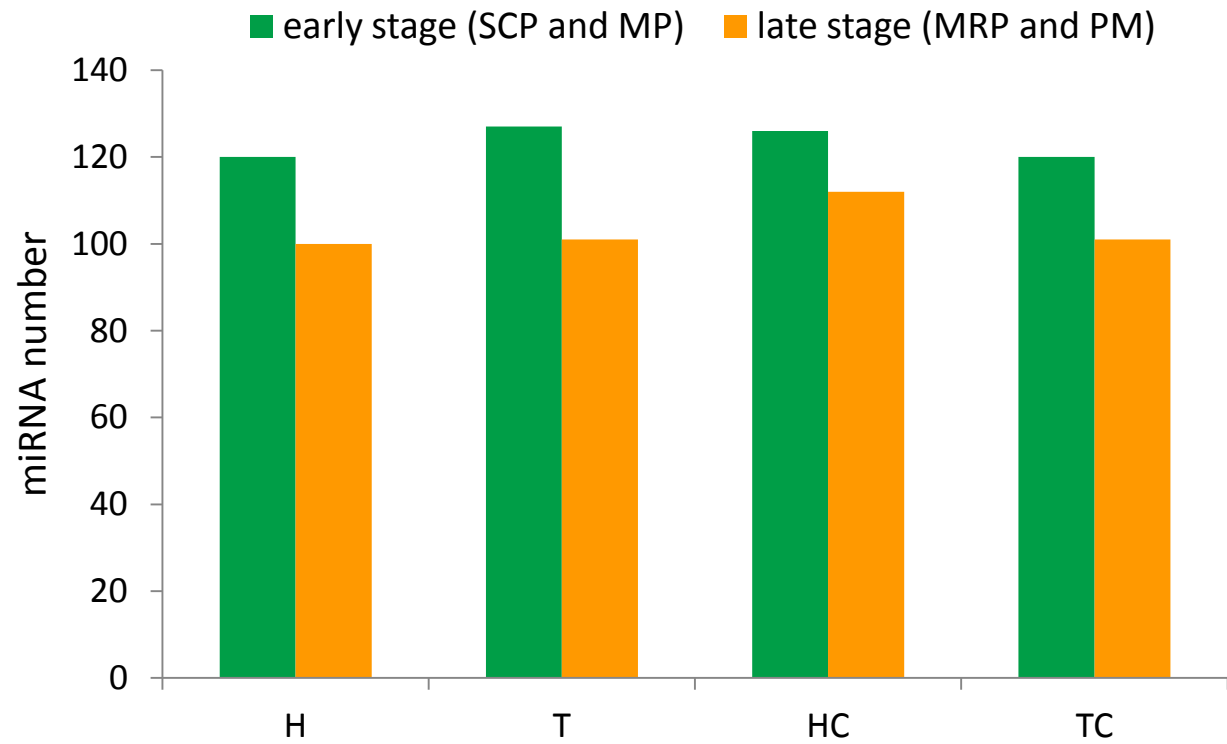

Supplement: Supplementary file 1 [file ijms-21-01280-s001.zip › Supplementary files/Supplementary Figure S5.pdf]

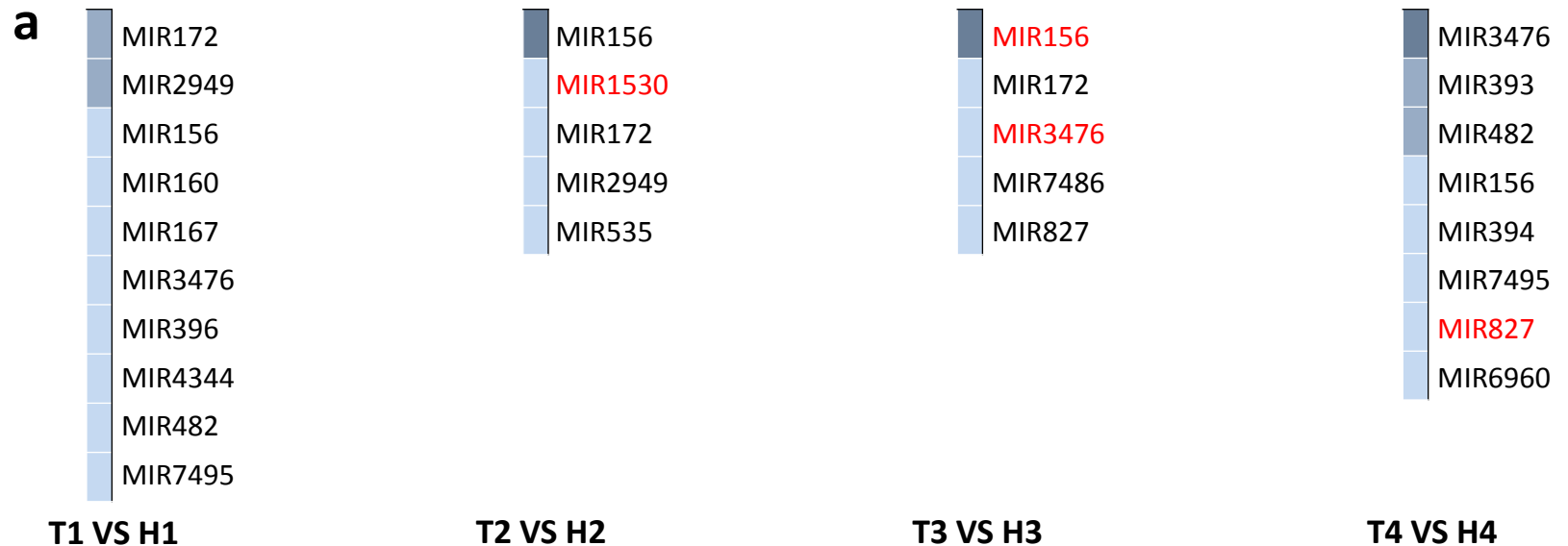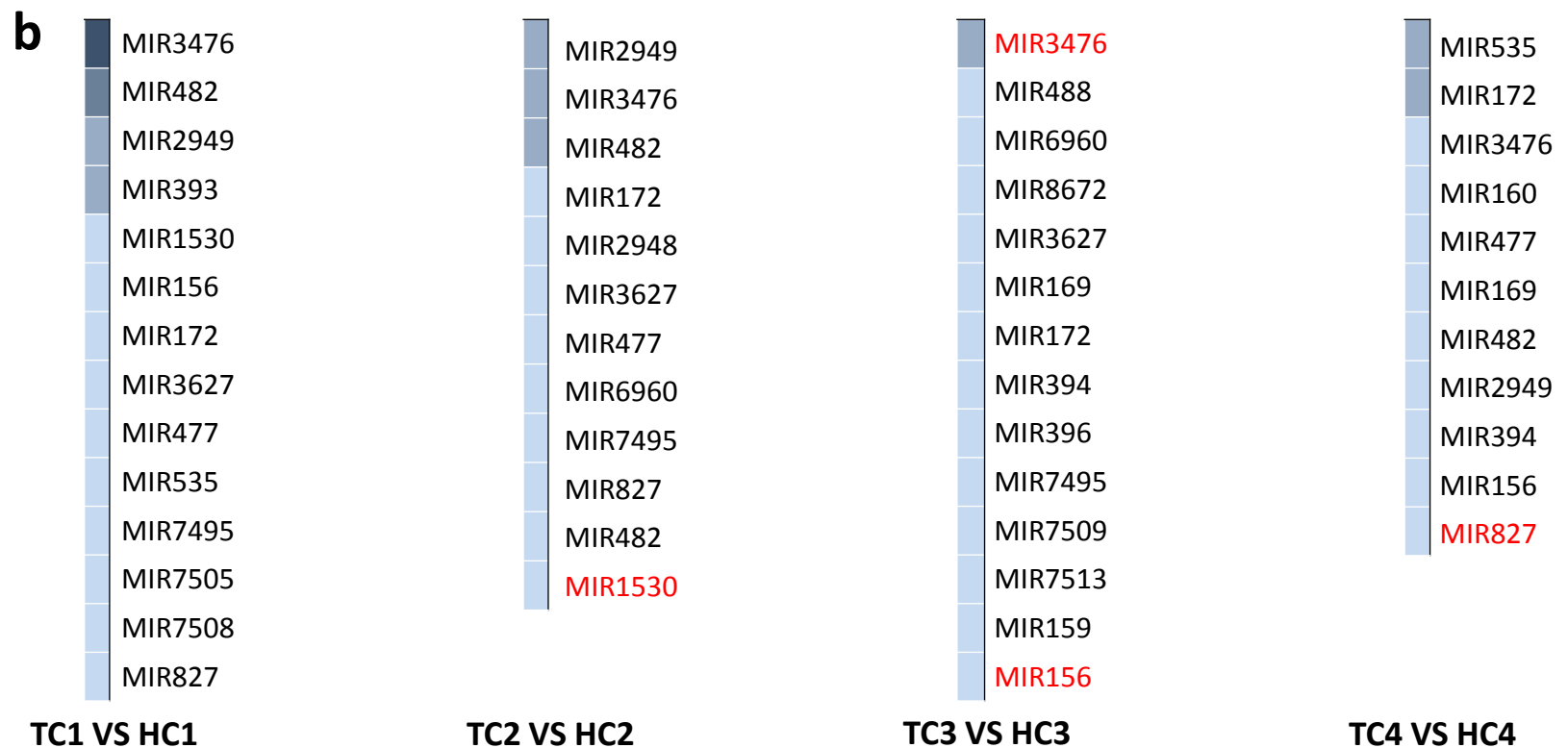

Supplement: Supplementary file 1 [file ijms-21-01280-s001.zip › Supplementary files/Supplementary Figure S6.pdf]

**HLY11 (H)**

**TS18 (T)**

**Normal temperature**

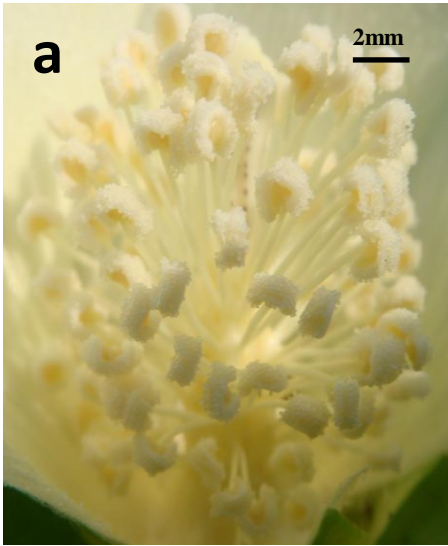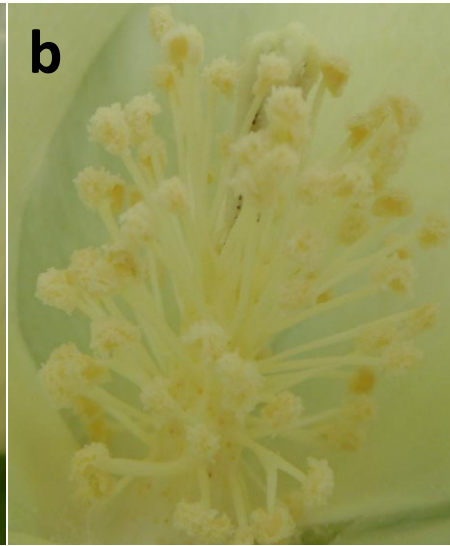

**High temperature**

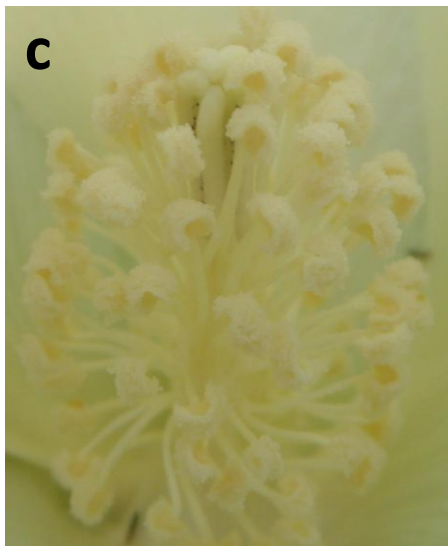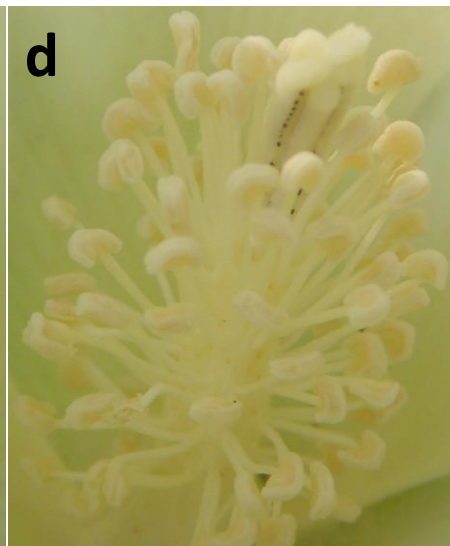

Supplement: Supplementary file 1 [file ijms-21-01280-s001.zip › Supplementary files/Supplementary Figure S7.pdf]
